# Impact of Opioid Use on Duration of Time Loss After Work-Related Lower Limb Injury

**DOI:** 10.1007/s10926-022-10048-5

**Published:** 2022-06-03

**Authors:** Katrina Szucs, Shannon E. Gray

**Affiliations:** 1 School of Public Health and Preventive Medicine, Monash University, 553 St Kilda Rd, Melbourne, VIC 3004 Australia; 2 Healthy Working Lives Research Group, School of Public Health and Preventive Medicine, Monash University, 553 St Kilda Rd, Melbourne, VIC 3004 Australia

**Keywords:** Lower extremity, Worker’s compensation, Injury, Opioid

## Abstract

*Purpose* This study sought to determine patterns of opioid use among workers with a compensated lower limb injury, factors associated with opioid use, and how opioid use is associated with time loss duration. *Methods* Claims and medication data were provided by the workers’ compensation regulator of Victoria, Australia, for claims lodged 2008–2018 from workers aged 15+ years with a lower limb injury. Descriptive statistics showed the number and prevalence of each opioid type (weak/strong) by demographic, claim and injury predictors. Binary and multinomial logistic regression determined the likelihood of any opioid use, and use of strong, weak or a combination of strong and weak opioids by predictors. Cox regression determined the effect of each opioid type on duration of time loss, controlling for predictors. *Results* There were 51,334 claims and of these 23.6% were dispensed opioids (9.2% for strong opioids only, 6.6% for weak opioids only and 7.8% for a combination). Weak opioids, on average, were dispensed 15 days earlier than strong opioids. Time loss claims and workers with fractures or hip injuries were most likely to be dispensed opioids. All opioids were associated with increased duration of time loss, with those dispensed both weak and strong opioids having the longest duration of time loss. *Conclusions* Any opioid use was associated with longer time loss duration, with increasing opioid strength having a greater effect. Review of pain management methods should be undertaken to reduce opioid use, which may have a positive impact on duration of time loss and long-term function.

## Introduction

It was estimated that in 2013, workplace injuries cost the Australian economy $61.8 billion per year, approximately 4.1% of Australia’s Global Domestic Product; most of which is borne by the injured worker [1]. Of all serious compensated workplace injuries that result in at least 1 week of absence from work, around a quarter are for lower limb injuries [2]. Of these, the knee and ankle were most commonly injured [2]. Lower limb injuries, especially those affecting the knee and ankle, are a substantial contributor to the economic burden of workplace injury to the Australian economy.

In Australia, the vast majority of workers are covered for work-related injury through compulsory workers’ compensation insurance regulated by state, territory and commonwealth government authorities. SafeWork Australia, a national policy body for workers’ compensation, has been collating and reporting on workers’ compensation statistics for over 20 years, providing insight into compensation trends in the Australian population [2]. Whilst the overall incidence rate of workers’ compensation claims decreased between 2000 and 2019, the duration of time loss (absence from work) for such claims is increasing [2]. As a result, the overall burden of workplace injuries is not decreasing among the Australian population.

Return to work is an important rehabilitation goal for workers and is an indicator of functional recovery after a workplace injury [3–7]. To support recovery for those injured at work, workers’ compensation systems can provide income support benefits whilst workers are unable to work and pay for healthcare expenses, including medicines, with the end goal a successful and sustained return to work.

Analgesics are medicines used to relieve pain in a diverse range of circumstances. To help manage the pain associated with a work-related injury, analgesia can be paid through workers’ compensation. The choice of analgesia normally depends on the nature and severity of the injury [8–11]. Simple analgesics such as paracetamol and non-steroidal anti-inflammatory drugs (NSAIDs) are widely used for the treatment of mild to moderate pain after injury, whereas stronger analgesics such as opioids are indicated for severe acute pain or cancer-related pain [10, 12, 13].

Opioids are commonly prescribed for management of work-related injuries and are being increasingly prescribed for the management of chronic, non-cancer related pain among the population [9, 13]. Opioid use among injured workers have been associated with delayed functional recovery and increased duration of time loss. Previous studies have identified possibly contributory factors for this association. Injury and pain severity may confound the association between opioid use and duration of time loss, however this effect is yet to be quantified [4, 5, 7, 9, 14–17]. Workers who are prescribed opioids have a higher rate of psychological disorders, which is thought to be related to longer duration of disability and higher likelihood of legal litigations surrounding their claim [18, 19]. The reverse is also true, that those with pre-existing mental health disorders are at increased risk of opioid use due to their disorder [20]. Increased stressful interactions with the workers compensation system has also been found to increase psychological distress in the worker, possibly increasing the risk of opioid misuse and may impact successful return to work [18, 21].

Whilst there are studies that investigated the impact of opioid use on duration of time loss, no known articles have explored this relationship following a work-related lower-limb injury. Prior studies have focused either on how work-related injuries in general or on how low back pain affected duration of time loss, and few have been conducted in the Australian workers’ compensation context [7, 9, 14, 15, 17]. To broaden our understanding of how opioid use affects recovery, it is necessary to investigate other types of conditions and bodily locations of injury to determine any differences. As duration of time loss among injured Australian workers’ is increasing, investigation into factors affecting recovery and successful return to work is necessary [2].

This study aims to determine the factors associated with opioid use after work-related lower limb injury and how opioid use affects duration of time loss. Findings will contribute to broadening understanding of factors affecting duration of time loss and functional recovery among workers with lower limb injuries.

## Methods

### Setting, Data Source and Cohort Selection

WorkSafe Victoria (WSV) regulates both the health and safety for workers and the workers’ compensation scheme in the state of Victoria, Australia. Claims and services data were provided by WSV through request. Cases were included if the claim was lodged between 01/01/2008 and 31/12/2018 (in order to capture a greater number of claims), the main injury was to the lower limb and the age at time of injury was 15 years or older. Lodgement of cases were limited to 2018 to allow for a maximum of 130 weeks follow-up, the maximum time WSV will pay income replacement. Claims data included information related to the claim (e.g. date of injury), the injured worker (e.g. age, sex, occupation), the employer (e.g. industry) and the injury (e.g. nature of injury).

Compensated opioids were extracted from the services dataset, including service date and description of the medicine. The medicine description included at least the generic name or brand name, and in some cases the dosage and type (e.g. capsule, tablet). The claims and services datasets had a one-to-many relationship (possible for multiple medication records per claim) and were linked by a deidentified claim number.

### Predictor Variables

Factors known to be associated with duration of time loss (compensated time away from work) were included as predictors for all analyses including age, gender, occupation, industry, remoteness, socioeconomic status, location and nature of injury (6).

Age at time of injury was categorized into 10-year age groups (e.g. 15–24 years) and 65 years and over. Sex (male/female) was taken directly from the provided claimant gender variable. Occupation was coded according to Australian and New Zealand Standard Classification of Occupations (ANZSCO) major group code [22]. Remoteness categories and socioeconomic status quintiles were derived using the workers’ residential postcode and mapped to the Australian Statistical Geographical Standard (ASGS) and Index of Relative Socio-economic Advantage and Disadvantage (IRSAD) respectively [23, 24]. Remoteness was collapsed into ‘Major Cities of Australia’ and ‘Regional/Remote’ due to smaller numbers in the regional to remote categories. Socioeconomic status was collapsed into the most disadvantaged quintile, most advantaged quintile and a combination of the middle three quintiles. Bodily location of injury and nature of injury was coded as per the Type of Occurrence Classification System (TOOCS 3.1) [25]. Location of injury was broken into ‘hip’, ‘thigh’, ‘knee’, ‘lower leg’, ‘ankle’ and ‘foot’ and nature of injury was collapsed into categories using the highest TOOCS 3.1 nature of injury code. Some injury types were broken down into lower order categories to enhance our understanding of nature of injury, and others were collapsed into higher order categories due to low numbers (see Table 2 for breakdown).

### Outcomes

Medication type, extracted from the medicine description variable, was mapped to the Anatomical Therapeutic Chemical Classification system and categorised into weak or strong opioids (see Table 1 for breakdown) [26]. The first outcome was whether an injured worker was dispensed an opioid. The second outcome was whether a worker had been dispensed weak, strong, weak and strong opioids, or no opioids.

**Table 1:** Descriptive statistics of dispensed opioids

	N workers dispensed opioid*	N total opioid doses dispensed	Mean times opioid dispensed per worker (SD)	Median time to first opioid dispensed (IQR)
Strong opioid	12,708	99,980	7.9 (29.9)	80 (9, 256)
Buprenorphine	694	9052	13.0 (25.7)	120 (18, 463)
Fentanyl	138	1854	13.4 (26.5)	70 (4, 251)
Hydromorphone	52	1354	26.0 (49.8)	216 (27, 298)
Morphine	134	2020	15.1 (36.9)	20 (− 7, 357)
Oxycodone	7937	51,845	6.5 (25.3)	60 (4, 212)
Oxycodone and naloxone	2295	19,007	8.3 (21.3)	74 (11, 269)
Tapentadol	1458	14,848	10.2 (23.9)	154 (21, 439)
Weak opioid	8959	73,522	8.2 (20.0)	65 (4, 252)
Codeine and acetylsalicylic acid	13	112	8.6 (20.4)	277 (277, 448)
Codeine and ibuprofen	224	1733	7.7 (19.6)	66 (13, 441)
Codeine and paracetamol	5966	46,959	7.9 (22.0)	71 (8, 246)
Tramadol	2725	24,508	9.0 (23.3)	57 (0, 247)
Tramadol and paracetamol	31	210	6.8 (14.5)	41 (− 3, 223)

*Could be dispensed multiple types of opioids

The third outcome, duration of time loss, was defined using number of compensated days as provided by WSV.

### Data Analysis

Types of opioids dispensed including the number of each dispensed, number of workers dispensed each opioid and median days (with corresponding interquartile range) to first dispense were also tabulated for those with opioids. Characteristics of all injured workers and those who were dispensed at least one type of opioid were presented, including the number of claims, proportion of the cohort and prevalence of opioid use. For those with opioid use, the mean number of opioids dispensed was also presented by predictor variable (with corresponding standard deviation).

To determine the likelihood of any opioid use in the cohort (first outcome), binary logistic regression including all predictors was performed. Multinomial logistic regression using the second outcome as the dependent variable including all predictors was performed to determine the likelihood of each predictor being dispensed each type of opioid. Results are presented as odds ratios with corresponding 95% confidence intervals.

Cox regression using only time loss claims was used to determine the association of opioid use, using the derived second outcome and controlling for all previous predictors, with duration of time loss. Results are presented as hazard ratios with corresponding 95% confidence intervals. Statistical significance for all analyses was set at p < 0.05.

Occupation, remoteness and socioeconomic status had 19.4%, 0.3%, and 0.1% missing values. Multiple imputation was performed to substitute missing values with plausible values through five iterations. A sensitivity analysis was conducted comparing all regression output between the original data and the imputed data. Results varied minimally, therefore the imputed results are presented.

Analyses were conducted using SPSS (Chicago, U.S.A) and R Version 4.0.3 (Auckland, New Zealand).

## Results

Over the 10-year study period there were 51,334 lower limb injury claims included in analysis, 76% of which were time loss claims. Of all claims, 12,708 workers were dispensed at least one strong opioid dose and 8959 were dispensed at least one weak opioid dose (Table 1). Of the 23.6% of workers who were dispensed opioids, 9.2% were for strong opioids only (n = 4699), 6.6% for weak opioids only (n = 3395) and 7.8% for a combination (n = 4023). The most common opioids prescribed were oxycodone (7937 workers, 51,845 dispenses) and codeine with paracetamol (5966 workers, 46,959 dispenses), however the opioids with the highest number of opioid doses dispensed per worker were hydromorphone and morphine. The median time to first dispensing of strong opioids was 15 days greater than weak opioids.

Workers with burns, other disease claims, and the youngest and eldest age groups had the lowest mean number of opioids prescribed (Table 2). Hip and knee injuries had the highest prevalence of being dispensed opioids. Hip injuries had the most dispenses per worker and the knee and ankle had the least. The prevalence of opioid use was similar between occupations.

**Table 2:** Descriptive statistics of cohort

	N (in cohort)	% of cohort	Dispensed at least one opioid (N)	Prevalence of being dispensed opioids (%)	Mean number of opioids (SD)
Gender					
Male	34,752	67.7	8500	24.5	13.3 (44.3)
Female	16,582	32.3	3617	21.8	16.8 (49.8)
Age group					
15–24 years	5117	10.0	773	15.1	7.7 (38.1)
25–34 years	9194	17.9	1964	21.4	12.7 (46.2)
35–44 years	10,995	21.4	2744	25.0	18.0 (54.7)
45–54 years	14,088	27.4	3636	25.8	16.3 (49.9)
55–64 years	10,516	20.5	2693	25.6	11.7 (32.9)
65+ years	1424	2.8	307	21.6	7.7 (18.5)
Nature of injury					
Fractures	9453	18.4	2430	25.7	15.9 (47.5)
Wounds, lacerations, amputations and internal organ damage	6767	13.2	1179	17.4	16.7 (48.0)
Burn	529	1.0	63	11.9	7.6 (16.0)
Dislocation	1849	3.6	469	25.4	12.8 (51.9)
Trauma to joints and ligaments (NEC)	14,221	27.7	3325	23.4	14.7 (49.3)
Trauma to muscles and tendons (NEC)	5189	10.1	1101	21.2	11.5 (34.7)
Musculoskeletal and connective tissue diseases	12,449	24.3	3414	27.4	13.3 (44.2)
Other injuries	337	0.7	74	22.0	17.7 (38.2)
Other diseases and claims	540	1.1	62	11.5	6.7 (15.5)
Location of injury					
Ankle	10,043	19.6	1912	19.0	13.7 (39.6)
Foot and toes	7545	14.7	1167	15.5	16.8 (42.4)
Hip	2158	4.2	705	32.7	25.1 (71.3)
Knee	24,139	47.0	6816	28.2	12.3 (44.1)
Lower leg	5408	10.5	1080	20.0	16.8 (48.8)
Upper leg	2041	4.0	437	21.4	18.9 (50.2)
Occupation*					
Clerical and Administrative Workers	662	1.3	149	22.5	14.3 (45.8)
Community and Personal Service Workers	10,051	19.6	2507	24.9	13.1 (47.0)
Labourers	2190	4.3	503	23.0	18.1 (51.8)
Machinery Operators and Drivers	9094	17.7	2205	24.2	16.5 (48.8)
Managers	2161	4.2	558	25.8	12.8 (38.4)
Professionals	6245	12.2	1426	22.8	11.5 (36.3)
Sales Workers	6009	11.7	1321	22.0	16.9 (55.6)
Technicians and Traders Workers	4987	9.7	1234	24.7	11.4 (49.0)
Industry					
Accommodation and Food Services	1688	3.3	305	18.1	13.1 (40.1)
Administrative and Support Services	1960	3.8	381	19.4	11.7 (32.1)
Agriculture, Forestry and Fishing	1517	3.0	282	18.6	20.1 (57.2)
Arts and Recreation Services	2116	4.1	487	23.0	10.6 (53.1)
Construction	7909	15.4	2117	26.8	15.4 (50.4)
Education and Training	4065	7.9	874	21.5	10.4 (36.4)
Electricity, Gas, Water and Waste Services	691	1.3	149	21.6	11.2 (35.1)
Financial and Insurance Services	291	0.6	82	28.2	14.0 (42.6)
Health Care and Social Assistance	6162	12.0	1353	22.0	13.7 (38.5)
Information Media and Telecommunications	273	0.5	60	22.0	15.0 (42.9)
Manufacturing	6988	13.6	1719	24.6	15.5 (46.0)
Mining	245	0.5	59	24.1	17.8 (40.4)
Other Services	1216	2.4	297	24.4	16.9 (51.6)
Professional, Scientific and Technical Services	1042	2.0	273	26.2	8.5 (27.1)
Public Administration and Safety	4143	8.1	1107	26.7	10.8 (38.1)
Rental, Hiring and Real Estate Services	619	1.2	144	23.3	8.2 (15.2)
Retail Trade	2723	5.3	598	22.0	18.3 (53.2)
Transport, Postal and Warehousing	4528	8.8	1094	24.2	18.5 (54.3)
Wholesale Trade	3158	6.2	736	23.3	14.8 (54.6)
Socioeconomic Group**					
Most advantaged	10,280	20.0	2355	22.9	8.3 (27.6)
Middle three quintiles	33,292	64.9	8003	24.0	15.2 (50.4)
Most disadvantaged	7703	15.0	1741	22.6	18.5 (44.4)
Remoteness***					
Major Cities of Australia	36,486	71.1	8900	24.4	13.5 (45.7)
Regional/Remote Australia	14,690	28.6	3174	21.6	16.6 (46.8)
Claim Type					
Medical only	12,145	23.7	842	6.9	3.4 (16.5)
Time loss	39,189	76.3	11,275	28.8	15.1 (47.4)

Musculoskeletal and connective tissue diseases can include conditions such as arthritis, meninscus degeneration, synovitis, tendonitis, and others

*N = 9935 missing; **N = 59 missing; ***N = 158 missing

Participants with the lowest prevalence of opioid doses dispensed were aged between 15 and 24 years old, experienced burns, wounds, lacerations, amputations and internal organ damage or submitted medical only claims.

The most disadvantaged socioeconomic quintile had a higher mean number of opioids dispenses than the highest socioeconomic quintile and claimants in regional/remote Victoria had a higher mean number of opioid doses dispensed than those in major cities per worker. Time loss claims had a higher mean number of opioid doses dispensed than medical only claims.

Table 3 shows that females had 14% lower odds of being dispensed a strong opioid alone compared to males, but 11% higher odds of being dispensed a combination of strong and weak opioids during their claim. Opioid use was lowest in the youngest and oldest age groups. Fractures were most likely to be dispensed opioids. Musculoskeletal and connective tissue diseases were 16% more likely to be dispensed weak opioids than the reference group (trauma to the joints and ligaments). Fractures and injuries to the hip were more likely to be dispensed either strong opioids or a combination of strong and weak opioids. Only hip injuries had higher odds of being dispensed opioids than knee injuries.

**Table 3:** Logistic regression results investigating the association of opioid use with predictor variables

	Binary logistic regression (reference: no dispensed opioids)		Multinomial regression (reference: no dispensed opioids)					
			Strong		Weak		Strong and weak	
	OR (95% CI)	p-value	OR (95% CI)	p-value	OR (95% CI)	p-value	OR (95% CI)	p-value
Gender								
Female	0.99 (0.93, 1.05)	0.668	0.86 (0.79, 0.94)	0.001	1.04 (0.94, 1.15)	0.448	1.11 (1.01, 1.21)	0.032
Male	Reference group							
Age group								
15–24 years	0.56 (0.51, 0.61)	< 0.001	0.73 (0.64, 0.83)	< 0.001	0.45 (0.38, 0.53)	< 0.001	0.46 (0.40, 0.54)	< 0.001
25–34 years	0.82 (0.77, 0.88)	< 0.001	0.90 (0.81, 0.99)	0.025	0.72 (0.65, 0.81)	< 0.001	0.84 (0.76, 0.93)	0.001
35–44 years	0.97 (0.92, 1.04)	0.404	0.92 (0.84, 1.00)	0.058	0.99 (0.90, 1.09)	0.875	1.02 (0.93, 1.12)	0.612
45–54 years	Reference Group							
55–64 years	0.95 (0.89, 1.01)	0.088	1.07 (0.98, 1.17)	0.108	0.88 (0.79, 0.97)	0.009	0.88 (0.80, 0.97)	0.008
65+ years	0.70 (0.61, 0.81)	< 0.001	0.88 (0.73, 1.06)	0.181	0.60 (0.46, 0.77)	< 0.001	0.60 (0.48, 0.75)	< 0.001
Nature of injury								
Fractures	1.41 (1.32, 1.52)	< 0.001	1.68 (1.52, 1.86)	< 0.001	1.00 (0.87, 1.14)	0.991	1.38 (1.24, 1.54)	< 0.001
Wounds, lacerations, amputations and internal organ damage	0.83 (0.77, 0.90)	< 0.001	0.76 (0.67, 0.86)	< 0.001	1.05 (0.92, 1.19)	0.477	0.74 (0.65, 0.84)	< 0.001
Burn	0.69 (0.52, 0.91)	0.009	0.63 (0.40, 0.99)	0.045	1.16 (0.75, 1.78)	0.508	0.46 (0.28, 0.76)	0.002
Dislocation	1.09 (0.97, 1.23)	0.132	1.13 (0.96, 1.34)	0.139	1.03 (0.84, 1.25)	0.791	1.11 (0.92, 1.33)	0.264
Other diseases and claims	0.51 (0.38, 0.67)	< 0.001	0.46 (0.29, 0.72)	0.001	0.69 (0.44, 1.08)	0.105	0.42 (0.27, 0.67)	< 0.001
Trauma to muscles and tendons (NEC)	0.90 (0.83, 0.98)	0.015	0.93 (0.82, 1.05)	0.222	1.02 (0.89, 1.16)	0.825	0.79 (0.69, 0.90)	< 0.001
Musculoskeletal and connective tissue diseases	1.06 (1.00, 1.12)	0.062	1.05 (0.97, 1.15)	0.249	1.16 (1.06, 1.28)	0.002	0.97 (0.88, 1.06)	0.502
Other injuries	1.21 (0.92, 1.60)	0.173	1.02 (0.66, 1.59)	0.919	1.48 (0.97, 2.25)	0.068	1.19 (0.77, 1.83)	0.429
Trauma to joints and ligaments (NEC)	Reference group							
Location of injury								
Ankle	0.55 (0.51, 0.58)	< 0.001	0.51 (0.47, 0.56)	< 0.001	0.43 (0.38, 0.48)	< 0.001	0.73 (0.66, 0.81)	< 0.001
Foot and toes	0.40 (0.36, 0.43)	< 0.001	0.34 (0.30, 0.38)	< 0.001	0.36 (0.31, 0.41)	< 0.001	0.53 (0.47, 0.60)	< 0.001
Hip	1.29 (1.17, 1.43)	< 0.001	1.29 (1.12, 1.48)	< 0.001	0.71 (0.59, 0.86)	0.001	2.02 (1.76, 2.33)	< 0.001
Upper leg	0.76 (0.68, 0.85)	< 0.001	0.79 (0.67, 0.93)	0.005	0.51 (0.41, 0.64)	< 0.001	1.02 (0.85, 1.22)	0.827
Lower leg	0.62 (0.57, 0.67)	< 0.001	0.57 (0.50, 0.64)	< 0.001	0.43 (0.37, 0.50)	< 0.001	0.92 (0.81, 1.04)	0.179
Knee	Reference group							
Occupation								
Clerical and Administrative Workers	0.95 (0.79, 1.15)	0.607	0.93 (0.70, 1.24)	0.626	0.81 (0.57, 1.14)	0.226	1.12 (0.84, 1.50)	0.435
Technicians and Trades Workers	1.10 (1.00, 1.20)	0.051	1.08 (0.94, 1.24)	0.288	0.98 (0.85, 1.13)	0.797	1.23 (1.04, 1.45)	0.018
Labourers	1.03 (0.91, 1.16)	0.667	0.90 (0.73, 1.11)	0.304	0.98 (0.82, 1.18)	0.869	1.23 (1.02, 1.49)	0.031
Machinery Operators and Drivers	0.98 (0.91, 1.05)	0.552	0.91 (0.80, 1.03)	0.139	0.95 (0.82, 1.10)	0.477	1.10 (0.97, 1.24)	0.132
Managers	1.23 (1.10, 1.38)	< 0.001	1.29 (1.09, 1.52)	0.003	1.02 (0.81, 1.27)	0.885	1.37 (1.15, 1.63)	0.001
Professionals	1.06 (0.97, 1.15)	0.192	1.00 (0.87, 1.14)	0.946	1.04 (0.89, 1.20)	0.639	1.17 (1.01, 1.34)	0.030
Sales Workers	0.98 (0.90, 1.07)	0.613	0.94 (0.83, 1.06)	0.288	0.93 (0.80, 1.07)	0.299	1.09 (0.95, 1.24)	0.210
Community and Personal Service Workers	Reference group							
Industry								
Wholesale Trade	0.86 (0.77, 0.95)	0.004	0.82 (0.70, 0.96)	0.011	0.98 (0.82, 1.16)	0.785	0.81 (0.69, 0.95)	0.011
Administrative and Support Services	0.72 (0.63, 0.82)	< 0.001	0.73 (0.61, 0.89)	0.001	0.83 (0.67, 1.04)	0.100	0.61 (0.50, 0.76)	< 0.001
Agriculture, Forestry and Fishing	0.64 (0.55, 0.74)	< 0.001	0.53 (0.42, 0.67)	< 0.001	0.81 (0.63, 1.05)	0.110	0.64 (0.52, 0.80)	< 0.001
Arts and Recreation Services	0.92 (0.81, 1.04)	0.197	1.10 (0.93, 1.31)	0.244	0.85 (0.68, 1.07)	0.165	0.75 (0.61, 0.92)	0.006
Accommodation and Food Services	0.67 (0.58, 0.78)	< 0.001	0.74 (0.61, 0.92)	0.005	0.71 (0.55, 0.92)	0.010	0.56 (0.44, 0.71)	< 0.001
Education and Training	0.86 (0.77, 0.96)	0.008	1.00 (0.86, 1.17)	0.960	0.92 (0.76, 1.12)	0.411	0.67 (0.56, 0.80)	< 0.001
Electricity, Gas, Water and Waste Services	0.84 (0.69, 1.02)	0.079	0.90 (0.68, 1.19)	0.465	0.95 (0.68, 1.32)	0.747	0.69 (0.50, 0.96)	0.026
Financial and Insurance Services	1.29 (0.97, 1.71)	0.082	1.27 (0.84, 1.90)	0.254	1.26 (0.77, 2.04)	0.354	1.33 (0.87, 2.02)	0.184
Health Care and Social Assistance	0.72 (0.65, 0.80)	< 0.001	0.73 (0.63, 0.84)	< 0.001	0.82 (0.69, 0.96)	0.017	0.65 (0.55, 0.76)	< 0.001
Information Media and Telecommunications	0.84 (0.62, 1.15)	0.283	1.12 (0.76, 1.66)	0.568	0.85 (0.50, 1.47)	0.571	0.52 (0.29, 0.93)	0.026
Manufacturing	0.92 (0.85, 1.00)	0.038	0.83 (0.74, 0.93)	0.002	1.09 (0.96, 1.25)	0.183	0.89 (0.79, 1.01)	0.065
Mining	0.98 (0.72, 1.34)	0.909	1.16 (0.76, 1.78)	0.481	0.90 (0.50, 1.60)	0.707	0.84 (0.52, 1.38)	0.494
Other Services	0.88 (0.76, 1.02)	0.090	0.85 (0.69, 1.05)	0.127	0.96 (0.75, 1.22)	0.733	0.86 (0.68, 1.08)	0.196
Professional, Scientific and Technical Services	1.19 (1.01, 1.40)	0.035	1.40 (1.13, 1.74)	0.002	1.07 (0.80, 1.42)	0.642	1.04 (0.81, 1.34)	0.768
Public Administration and Safety	1.01 (0.92, 1.12)	0.815	1.01 (0.88, 1.16)	0.905	1.14 (0.97, 1.34)	0.109	0.92 (0.79, 1.07)	0.295
Rental, Hiring and Real Estate Services	0.87 (0.71, 1.07)	0.192	0.88 (0.65, 1.18)	0.385	0.79 (0.54, 1.15)	0.226	0.93 (0.68, 1.25)	0.620
Retail Trade	0.78 (0.70, 0.88)	< 0.001	0.72 (0.61, 0.86)	< 0.001	0.91 (0.75, 1.10)	0.313	0.75 (0.63, 0.90)	0.002
Transport, Postal and Warehousing	0.88 (0.80, 0.96)	0.006	0.78 (0.67, 0.90)	0.001	0.98 (0.83, 1.16)	0.858	0.90 (0.78, 1.04)	0.149
Construction	Reference group							
Socioeconomic group								
Most disadvantaged	0.96 (0.90, 1.03)	0.244	0.91 (0.83, 1.00)	0.044	0.99 (0.89, 1.10)	0.811	1.01 (0.91, 1.11)	0.915
Most advantaged	0.96 (0.90, 1.01)	0.136	1.00 (0.92, 1.08)	0.963	0.95 (0.86, 1.04)	0.282	0.91 (0.83, 1.00)	0.052
Middle 3 quintiles	Reference Group							
Remoteness								
Regional/Remote Australia	0.81 (0.77, 0.85)	< 0.001	0.80 (0.74, 0.86)	< 0.001	0.68 (0.63, 0.75)	< 0.001	0.92 (0.85, 1.00)	0.049
Major Cities of Australia	Reference group							
Claim type								
Medical only	0.18 (0.16, 0.19)	< 0.001	0.18 (0.16, 0.20)	< 0.001	0.30 (0.27, 0.34)	< 0.001	0.07 (0.06, 0.09)	< 0.001
Time loss	Reference Group							

Managers had 23% higher odds than the reference (Community and Personal Service Workers) to be dispensed opioids. Compared to the Construction industry, all except Professional, Scientific and Technical Services had significantly lower odds of opioid use. Financial and Insurance Services had higher odds (OR 1.29) however did not reach statistical significance of p < 0.05. There was no statistically significant relationship between odds of opioid use and socioeconomic status, however those living in regional/remote Victoria had 19% lower odds than those in major cities.

Medical only claims had 82% lower odds of opioid use compared to time loss claims.

Table 4 shows that females, those aged 56 years and over, with fractures or hip injuries, and labourers had significantly longer durations of time loss. Dispensation of all types of opioids were associated with increased duration of time loss. It was found that as the strength of opioid increased (weak, strong to weak and strong, respectively) the median compensated days increased. A combination of strong and weak opioids was related to the longest duration of time loss, followed by strong opioids alone and weak opioids alone. It was not determined whether the strong and weak opioids were prescribed concurrently or at different time intervals.

**Table 4:** Cox regression results for predictors of duration of time loss

	Median compensated days (IQR)	HR (95% CI)	p-value
Gender			
Female	47 (17, 133)	0.86 (0.84, 0.89)	< 0.001
Male	42 (15, 120)	Reference	
Age group			
15–24 years	28 (11, 71)	1.43 (1.37, 1.48)	< 0.001
25–34 years	37 (14, 94)	1.20 (1.17, 1.24)	< 0.001
35–44 years	44 (16, 124)	1.05 (1.02, 1.08)	< 0.001
45–54 years	46 (16, 138)	Reference	
55–64 years	54 (19, 170.5)	0.89 (0.86, 0.91)	< 0.001
65+ years	67 (25, 191)	0.84 (0.79, 0.89)	< 0.001
Nature of injury			
Fractures	55 (26, 145)	0.84 (0.81, 0.86)	< 0.001
Wounds, lacerations, amputations and internal organ damage	27 (9, 88)	1.10 (1.06, 1.14)	< 0.001
Burn	18 (9, 37)	1.40 (1.26, 1.54)	< 0.001
Dislocation	43.5 (17, 120)	0.96 (0.9, 1.01)	0.117
Other diseases and claims	24 (10, 77)	1.07 (0.96, 1.18)	0.229
Trauma to muscles and tendons (NEC)	40 (14, 102)	1.04 (1, 1.08)	0.033
Musculoskeletal and connective tissue diseases	49 (17, 145)	0.96 (0.93, 0.98)	0.001
Trauma to joints and ligaments (NEC)	41 (13, 121)	Reference	
Other injuries	41 (16, 189)	0.88 (0.76, 1.01)	0.064
Location of injury			
Ankle	41 (15, 104)	1.09 (1.06, 1.12)	< 0.001
Foot and toes	36 (15, 95)	1.09 (1.06, 1.13)	< 0.001
Hip	98 (36, 370)	0.74 (0.7, 0.78)	< 0.001
Upper leg	34.5 (11, 136)	1.07 (1.01, 1.13)	0.02
Knee	47 (16, 131)	Reference	
Lower leg	38.5 (12, 120)	1.06 (1.02, 1.1)	0.005
Occupation			
Clerical and Administrative Workers	31 (10, 80)	1.20 (1.07, 1.33)	0.002
Technicians and Trades Workers	37 (14, 96)	1.06 (1.01, 1.1)	0.011
Labourers	55.5 (20, 178)	0.87 (0.83, 0.92)	< 0.001
Machinery Operators and Drivers	50 (18, 146)	0.93 (0.9, 0.96)	< 0.001
Managers	40 (15, 115)	1.04 (0.98, 1.11)	0.214
Professionals	35 (13, 95)	1.04 (0.99, 1.1)	0.099
Community and Personal Service Workers	41 (15, 112)	Reference	
Sales Workers	44 (16, 119)	0.99 (0.95, 1.03)	0.652
Industry			
Wholesale Trade	41 (15, 119)	1.13 (1.08, 1.19)	< 0.001
Administrative and Support Services	43 (13, 125)	1.04 (0.98, 1.1)	0.169
Agriculture, Forestry and Fishing	46 (16, 126)	0.99 (0.93, 1.05)	0.766
Arts and Recreation Services	37 (15, 101)	1.22 (1.15, 1.29)	< 0.001
Accommodation and Food Services	41 (15, 115)	1.07 (1, 1.14)	0.048
Education and Training	30 (11, 75)	1.56 (1.48, 1.65)	< 0.001
Electricity, Gas, Water and Waste Services	40 (14, 115)	1.19 (1.08, 1.3)	< 0.001
Financial and Insurance Services	27.5 (9.5, 69.5)	1.71 (1.47, 1.98)	< 0.001
Health Care and Social Assistance	50 (19, 132)	1.21 (1.16, 1.27)	< 0.001
Information Media and Telecommunications	37 (14, 82)	1.31 (1.13, 1.52)	< 0.001
Manufacturing	46 (16, 136)	1.08 (1.04, 1.12)	< 0.001
Mining	41 (13, 90)	1.23 (1.07, 1.42)	0.005
Other Services	44 (14, 135)	1.09 (1.01, 1.17)	0.018
Professional, Scientific and Technical Services	35.5 (12, 98)	1.41 (1.29, 1.53)	< 0.001
Public Administration and Safety	37 (12, 107)	1.38 (1.31, 1.44)	< 0.001
Rental, Hiring and Real Estate Services	39 (15, 106)	1.32 (1.19, 1.45)	< 0.001
Retail Trade	48 (17, 135)	1.09 (1.03, 1.14)	0.002
Transport, Postal and Warehousing	51 (18, 156)	1.05 (1.01, 1.1)	0.025
Construction	47 (18, 136)	Reference	
Socioeconomic Group			
Most disadvantaged	48 (16, 146)	0.94 (0.91, 0.96)	< 0.001
Middle 3 quintiles	44 (16, 126)	Reference	
Most advantaged	38 (14, 101)	1.09 (1.06, 1.12)	< 0.001
Remoteness			
Major Cities of Australia	44 (16, 124)	Reference	
Regional/Remote Australia	43 (15, 124)	1.00 (0.98, 1.03)	0.909
Opioid Type			
Strong	103 (39, 295)	0.46 (0.45, 0.48)	< 0.001
Weak	72 (26, 197)	0.57 (0.55, 0.59)	< 0.001
Strong and weak	331 (98, 636)	0.28 (0.27, 0.29)	< 0.001
No opioids	31 (12, 73)	Reference	

*IQR* is interquartile range, *HR* hazard ratio; HR < 1 means longer duration of time loss

## Discussion

This study sought to determine the characteristics of workers with lower limb injuries who were dispensed opioids to aid their recovery, and how this opioid use affected duration of time loss. Dispensation of opioids was associated with duration of time loss, with increasing strength related to longer durations of time loss.

Oxycodone, codeine with paracetamol, oxycodone with naloxone and tramadol were the most dispensed opioids. Previously an over-the counter drug, codeine recently became a Schedule 4 prescribed medicine due to a change in legislation to combat the rising rates of use in the population [27]. In Australia, an increasing number of opioid-related deaths, particularly among those over 30 years of age, are commonly being observed with oxycodone, codeine and tramadol [28]. With a high number of dispenses of these opioids in the workers compensation population, effective strategies to monitor opioid use to therefore reduce opioid-related harm and deaths should be considered.

Workers who were between male, aged 45 and 64 years or had submitted time loss claims had higher likelihood of being dispensed opioids. This is approximately 5 to 10 years older on average than previous findings in the literature [4, 14]. Cohort differences in the location and nature of injuries that differed from other studies may explain this variation. Alternatively, this result may have been affected by differences in the way age was analysed in our study compared to other studies, which included age as a continuous variable rather than categorise them.

Fractures, dislocations and injuries involving the joints and ligaments had a higher likelihood of opioid use and extended duration of time loss. This aligns with findings from existing literature and may be related to the longer healing process for traumatic injuries involving bone, joints and the surrounding soft tissues [4].

New insights into the differences in opioid prescription patterns based on location and nature of injury within the lower limb were found. Whilst injuries to the hip accounted for a low number of claims (4.2%), the proportion of opioid use and duration of time loss was high. Injuries involving the hip or knee were more likely than any other region in the lower limb to be prescribed opioids and were associated with increased durations of time loss. Hip and knee injuries exerted a strong effect on duration of time loss and utilization of opioids in workers. This research was the first known to investigate the effect of opioid use on duration of time loss in lower limb injuries specifically, which helps to fill the knowledge gap on how particular joint and ligament injuries affect opioid use and duration of time loss. More detailed investigation into the types of joint and ligament injuries associated with opioid use and extended duration of time loss will provide further insight for future studies.

A proposed reason for the high likelihood of opioid use and extended duration of time loss among workers with hip injuries is due to the anatomical location of the hip and pelvis. Due to the bodily location of a hip, there are challenges to successful offloading which is important for pain management and recovery. This may increase the likelihood of experiencing pain and sleep disturbances due to pain, which may explain higher utilization of opioids compared to other bodily locations such as the foot and toes. Workers with hip injuries may benefit from tailored support focusing on physical rehabilitation and occupational therapy to reduce the need for prolonged opioid use to treat persistent pain.

This study found no statistically significant relationship between socioeconomic status and the likelihood of being prescribed an opioid among the cohort, however it did find that there was an association between socioeconomic status and duration of time loss. This diverges from existing literature conducted in Canadian and United States jurisdictions which have varied results. One study found that the highest socioeconomic group were more likely to be prescribed opioids, whereas another found that the highest proportion of opioid use was within the lowest socioeconomic group [4, 9]. A possible reason for this difference could be due to the differences in defining income. Similar to Carnide et al. [9], this study used average postcode income to calculate socioeconomic status, whereas Gross et al. [4] used individual annual earnings. Findings from this study indicate that whilst opioid use does not consistently follow a socioeconomic gradient, with the most disadvantaged groups being most likely to use opioids, it is associated with extended duration of time loss. The reason for this effect should be investigated to further our understanding of the specific socioeconomic factors that affect duration of time loss. There were longer durations of time loss for those working in manual labour jobs, which would be more represented by workers from lower socioeconomic areas, likely due to the inability to accommodate return to work in these roles. This presents an opportunity for future exploration into the association of occupation based on socioeconomic status, and subsequent time loss.

Similarities in workers’ compensation systems between the Unites States, Canada and Australia suggests that findings are applicable to Australian workers’ compensation populations. Although Australia has a lower prevalence of opioid use compared to other countries [5], the regulation of opioid prescription should be carefully monitored based on their impact on functional recovery. Existing clinical guidelines provide evidence-based procedures for prescribers to follow to prevent inappropriate prescribing patterns and misuse following an injury. When prescribing opioids for chronic, non-malignant pain clinical guidelines currently indicate that non-pharmacological methods and non-opioid analgesia should be exhausted before trialing opioids [29]. When opioid analgesia is to be prescribed, it should be reviewed every 4–6 weeks to ensure that the following conditions are satisfied: adequate analgesia, improvement in function, absence of problematic behaviours and adverse effects from the medication [29]. Opioids should be ceased if the patient does not meet any one of the conditions. Results from this study show that opioids are not usually prescribed within the first 30 days of the claim, which may indicate that median first opioid prescription adheres to clinical guidelines for this cohort.

Interesting patterns on the type and timing of opioid prescription were found among the cohort. Findings from this study indicate that on average weak opioids were prescribed 15 days earlier in the claim than strong opioids. This suggests opioids are being prescribed for chronic, non-cancer pain in some cases and therefore should be carefully monitored. Previous literature investigating the effect of early (within 30 days of claim onset) versus late opioid prescription (> 30 days) on duration of time loss, found that early prescription of opioids was associated with prolonged claim duration and increased the likelihood of late opioid prescription [15, 16]. In line with previous literature which found a dose–response relationship, our study found that with increasing opioid strength there was a stronger effect on duration of time loss [15, 16]. This suggests that the strength of the opioid prescribed may be more influential than timing on the duration of time loss. Whilst timing of opioid was not included in Cox regression modelling, this study found that strong or combination opioid use was more strongly associated with increased duration of time loss than weak opioids (compared to no opioid use). This provides a basis to the hypothesis that strength of prescription is an important contributing factor to duration of time loss. This relationship between opioid strength and time loss is likely due to injured workers generally not being prescribed large doses of strong opioids initially due to lack of opioid tolerance and risk of respiratory depression, but with increased tolerance there is increased opioid dose. Consequently, increasing opioid dose takes time, evidenced by weaker opioids generally being prescribed 2 weeks earlier than stronger opioids in this study.

Chronicity of injury in workers with extended time loss claims may increase their vulnerability to mental health concerns. Due to the high burden of disease for mental health disorders among the general Australian population, it is important to develop a stronger understanding of the intersection between opioid use, duration of time loss and mental health disorders [30]. A US study found that opioid use was significantly higher among those with psychological disorders, therefore improving pain management is vital to reducing opioid usage in all populations, not just work-related injury [21].

In Australia, around 74% of work loss costs are being borne by the worker, which is a trend that is increasing over time mainly due to wage growth and upstream effects on human capital costs [1]. The resultant loss of income for injured workers increases the financial burden on the individual and can contribute to emotional stress related to the claim [21]. Although number of claims in Australia is reducing, duration of time loss for claims is increasing. As it was found that opioid use in all strengths extended duration of time loss it is recommended that further investigation into the causative factors behind this relationship are investigated. Severity of injury, pain, or psychological factors may have influenced the relationship between opioid use and duration of time loss, however, was not available for inclusion as a predictor variable. The duration of time loss for each worker may be reflective of injury severity which would benefit from validation in future studies. Further investigation into the effect of injury severity or pain on opioid use and claim duration is also warranted which would be best investigated using qualitative study methods or detailed participant interviews for data collection.

### Strengths and Limitations of the Study

This is the first known study to investigate the effect of opioid use on duration of time loss in those with lower limb injuries, with results highlighting significant variation in how different types of opioid use can affect duration of time loss. This study also expanded our understanding of who is more likely to use opioids (including nature of injury and injury location).

The study found a significant association between opioid use and duration of time loss; however, we were unable to control for severity of pain and injury because this study used administrative claims data. Additionally, time loss data presented in this study is cumulative compensated duration of absence, and does not consider uncompensated time away from work or other return to work measures due to the administrative nature of the data. Data used to prepare this paper was collected for administrative purposes and therefore some data quality issues may be present. During data collection, it was noted that data pertaining to the payment of the claim was most complete and more data were missing from variables relating to demographics and injury specifics. Usually, data within this database is coded by a claims manager, so the accuracy of such information is reliant on their input and interpretation of medical reports.

As Victoria has a 2-week excess period (or first ten working days) whereby the employer is responsible for income replacement and medical expenses up to a nominated amount before the insurer takes over, some inaccuracies in duration of time loss are assumed. Time loss claims less than 10 working days may not be captured in the dataset, and the true duration of time loss for claims lodged with WorkSafe Victoria may be longer than stated. The accuracy of compensated duration of time loss is unknown as the data is supplied by WorkSafe Victoria. Further, opioid use during the excess period is unlikely to have been fully captured in this study.

## Conclusion

Workers with fractures, hip injuries and time loss claims were most likely to use opioids after injury. Overall, opioid use was associated with increased duration of time loss and increasing opioid strength exerted a greater effect on duration of time loss. Oxycodone, codeine with paracetamol and tramadol were the most common opioids dispensed. Given the association of these medicines with the current opioid crisis and effect on functional recovery in workers compensation settings, stringent monitoring and regulation of dispensation is required. Non-pharmacological strategies to reduce the need for and duration of opioid use after injury should be investigated.

## Data Availability

Data is available from WorkSafe Victoria through request.

## Code Availability

Analyses were conducted using SPSS (Chicago, U.S.A) and R Version 4.0.3 (Auckland, New Zealand).

## References

[1] Safe Work Australia. The cost of work-related injury and illness for Australian employers, workers and the community: 2012–13. Canberra ACT: Safe Work Australia; 2015. p. 47.

[2] Safe Work Australia. Australian workers’ compensation statistics 2018–19. Canberra ACT: Safe Work Australia; 2021. p. 64.

[3] Pransky G, Gatchel RJ, Linton S, Loisel P (2005). Improving return to work research. J Occup Rehabil.

[4] Gross DP, Stephens B, Bhambhani Y, Haykowsky M, Bostick GP, Rashiq S (2009). Opioid prescriptions in Canadian workers’ compensation claimants: prescription trends and associations between early prescription and future recover. Spine.

[5] Tao X, Lavin RA, Yuspeh L, Weaver VM, Bernacki EJ (2015). The association of the use of opioid and psychotropic medications with workers’ compensation claim costs and lost work time. JOEM.

[6] Collie A, Lane TJ, Hassani-Mahmooei B (2016). Does time off work after injury vary by jurisdiction? A comparative study of eight Australian workers’ compensation systems. BMJ Open.

[7] Lavin RA, Tao X, Yuspeh L, Kalia N, Bernacki EJ (2016). Relationship between opioid prescribing patterns and claim duration and cost. JOEM.

[8] Yang J, Bauer B, Wahner-Roedler D, Chon T, Xiao L (2020). The modified WHO analgesic ladder: is it appropriate for chronic non-cancer pain?. J Pain Res.

[9] Carnide N, Hogg-Johnson S, Koehoorn M, Furlan AD, Côté P (2019). Relationship between early prescription dispensing patterns and work disability in a cohort of low back pain workers’ compensation claimants: a historical cohort study. Occup Environ Med.

[10] Ridderikhof M, Saanen J, Goddjin H, Van Dieren S, Etten-Jamaludin F, Lirk P (2019). Paracetamol versus other analgesia in adult patients with minor musculoskeletal injuries: a systematic review. Emerg Med J.

[11] Rosenbloom M, Khan S, McCartney C, Katz J (2013). Systematic review of persistent pain and psychological outcomes following traumatic musculoskeletal injury. J Pain Res.

[12] Hung KKC, Graham CA, Lo RSL, Leung YK, Leung LY, Man SY (2018). Oral paracetamol and/or ibuprofen for treating pain after soft tissue injuries: single centre double-blind, randomised controlled clinical trial. PLoS ONE.

[13] Mai J, Franklin G, Tauben D (2015). Guideline for prescribing opioids to treat pain in injured workers. Phys Med Rehabil Clin N Am.

[14] Steenstra IA, Busse JW, Tolusso D, Davilmar A, Lee H, Furlan AD (2015). Predicting time on prolonged benefits for injured workers with acute back pain. J Occup Rehabil.

[15] Webster BS, Verma SK, Gatchel RJ (2007). Relationship between early opioid prescribing for acute occupational low back pain and disability duration, medical costs, subsequent surgery and late opioid use. Spine.

[16] Busse JW, Ebrahim S, Heels-Ansdell D, Wang L, Couban R, Walter SD (2015). Association of worker characteristics and early reimbursement for physical therapy, chiropractic and opioid prescriptions with workers’ compensation claim duration, for cases of acute low back pain: an observational cohort study. BMJ Open.

[17] Tao X, Lavin RA, Yuspeh L, Weaver VM, Bernacki EJ (2015). Is early prescribing of opioid and psychotropic medications associated with delayed return to work and increased final workers’ compensation cost?. JOEM.

[18] Dersh J, Mayer TG, Gatchel RJ, Polatin PB, Theodore BR, Mayer EAK (2008). Prescription opioid dependence is associated with poorer outcomes in disabling spinal disorders. Spine.

[19] Faour M, Anderson JT, Haas AR, Percy R, Woods ST, Ahn UM (2018). Preoperative opioid use a risk factor for poor return to work status after single-level cervical fusion for radiculopathy in a workers’ compensation setting. Clin Spine Surg.

[20] Davis MA, Lin LA, Liu H, Sites BD (2017). Prescription opioid use among adults with mental health disorders in the United States. J Am Board Fam Med.

[21] Collie A, Sheehan L, Lane TJ, Iles R (2020). Psychological distress in workers’ compensation claimants: prevalence, predictors and mental health service use. J Occup Rehabil.

[22] Australian Bureau of Statistics. 1220.0—ANZSCO—Australian and New Zealand standard classification of occupations, 2013, version 1.2. (Australian Bureau of Statistics, Canberra, 2013).

[23] Australian Bureau of Statistics. 1270.0.55.006—Australian Statistical Geography Standard (ASGS): correspondences, July 2011. Canberra: Australian Bureau of Statistics; 2012.

[24] Australian Bureau of Statistics. Postal area, indexes, SEIFA (Socio-Economic Indexes for Areas) 2011. In: Census of population and houseing: socio-economic indexes for areas (SEIFA), Australia, 2011, Canberra; 2013.

[25] Australian Safety and Compensation Council. Type of occurrence classification system. Canberra ACT: Australian Safety and Compensation Council; 2008. p. 292.

[26] ATC/DDD Index 2021 [Internet]. Who collaborating centre for drug statistics methodology; 2020. https://www.whocc.no/atc_ddd_index/.

[27] Therapeutic Goods Administration. Current list of up-scheduled codeine containing products [Internet]. Department of Health—Therapeutic Goods Administration; 2018. https://www.tga.gov.au/community-qa/current-list-scheduled-codeine-containing-products.

[28] Karanges E, Blanch B, Buckley N, Pearson S-A (2016). Twenty-five years of prescription opioid use in Australia: a whole-of-population analysis using pharmaceutical claims. Br J Clin Pharmacol.

[29] Trescot AM, Boswell MV, Atluri SL, Hansen HC, Deer TR, Abdi S (2006). Opioid guidelines in the management of chronic non-cancer pain. Pain Physician.

[30] Slade T, Johnston A, Oakley Browne MA, Andrews G, Whiteford H (2009). 2007 National Survey of Mental Health and Wellbeing: methods and key findings. Aust NZ J Psychiatry.

